# Supplementary material for: Hybrid topological photonic crystals
Source: Nat Commun. 2023 Jul 25;14:4457. doi: 10.1038/s41467-023-40172-6 (PMC10368673; doi:10.1038/s41467-023-40172-6)
Supplement: Supplementary file 1 — Supplementary Information [file 41467_2023_40172_MOESM1_ESM.pdf]

Supplementary Information

Hybrid topological photonic crystals

Wang *et al.*

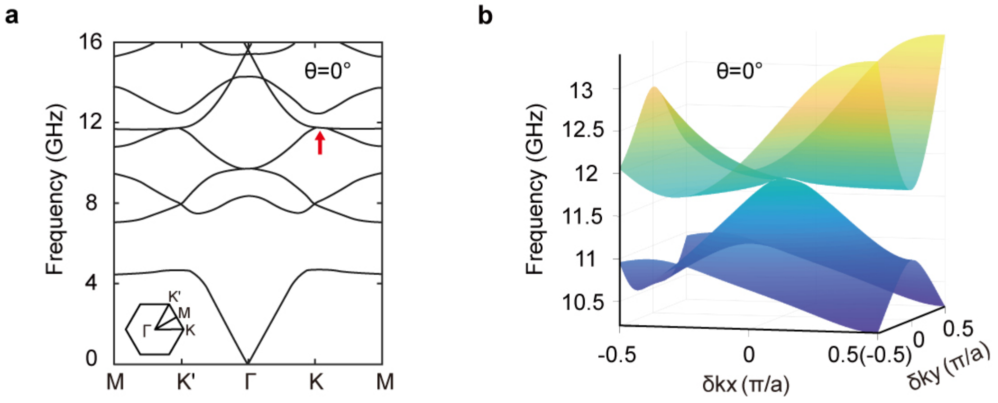

Supplementary Figure 1 | Band structure of HTPC with multiple Dirac points. a Photonic band structure of the HTPC with $\theta = 0^{\circ}$ in the absence of the external magnetic field. **b** The quadratic dispersion around Dirac point.

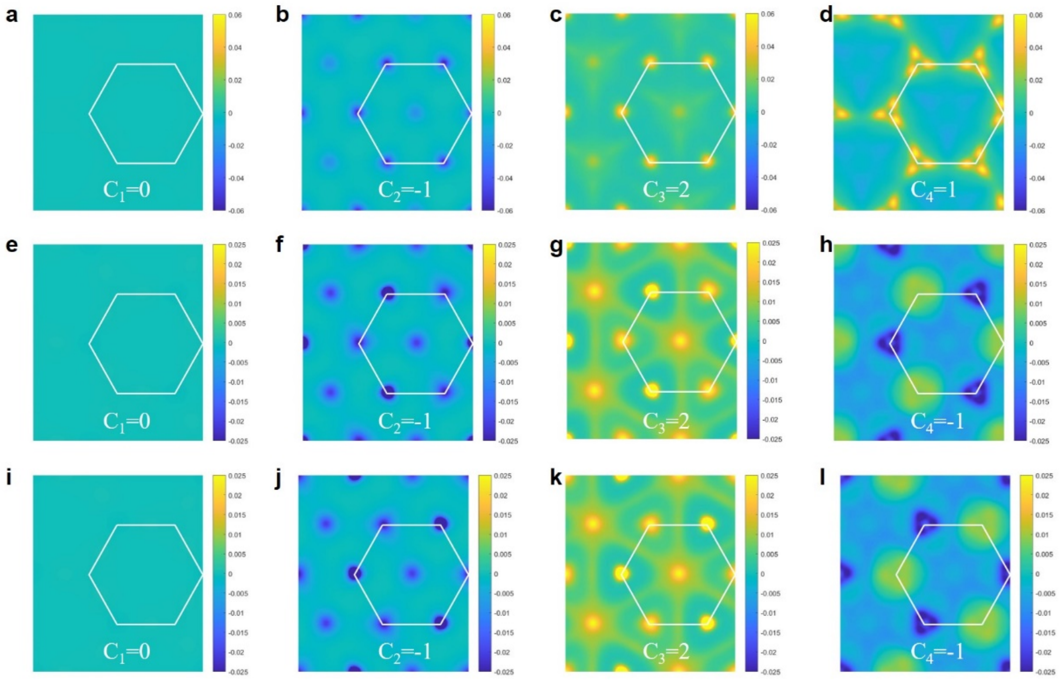

Supplementary Figure 2 | Band Chern number calculation via the integrating of the Berry curvature. **a-l** Berry curvature distributions of (**a,e,i**) the first, (**b,f,j**) the second, (**c,g,k**) the third, (**d,h,l**) the fourth band for HTPC with (**a-d**) $\theta = 0^{\circ}$, (**e-h**) $\theta = 30^{\circ}$ (i.e., HTPC1), (**i-l**) $\theta = 90^{\circ}$ (i.e., HTPC2). Note that the band Chern numbers, which are calculated by integrating the band Berry curvature, are also labeled. The first Brillouin zone is indicated by the white hexagon.

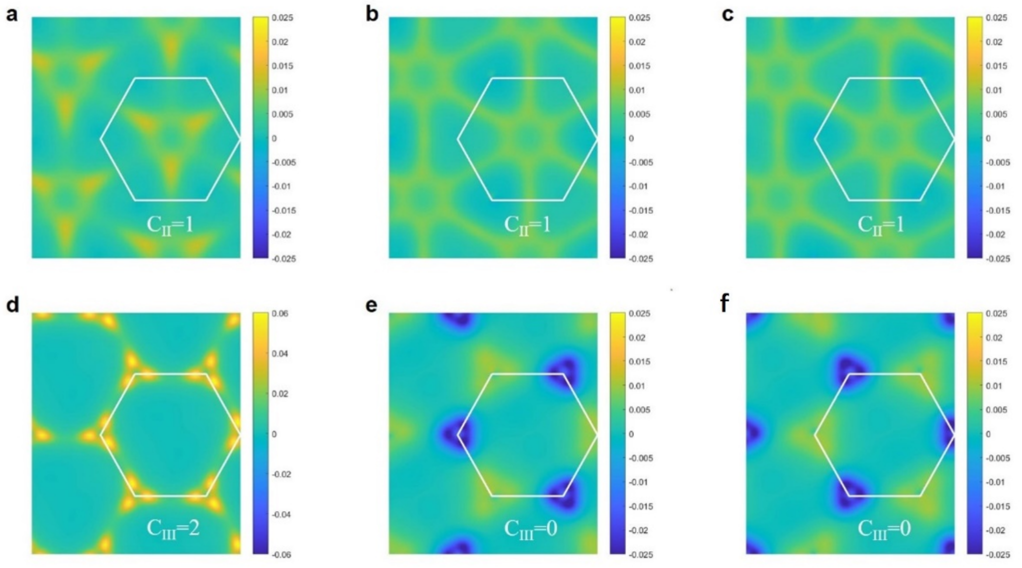

Supplementary Figure 3 | Gap Chern number calculation via the integrating of the Berry curvature. a-d Berry curvature distributions of (**a-c**) gap II and (**d-f**) gap II for HTPC with (**a,d**) $\theta = 0^{\circ}$, (**b,e**) $\theta = 30^{\circ}$ (i.e., HTPC1), (**c,f**) $\theta = 90^{\circ}$ (i.e., HTPC2). Note that the gap Chern numbers, which are calculated by integrating the gap Berry curvature, are also labeled. The first Brillouin zone is indicated by the white hexagon.

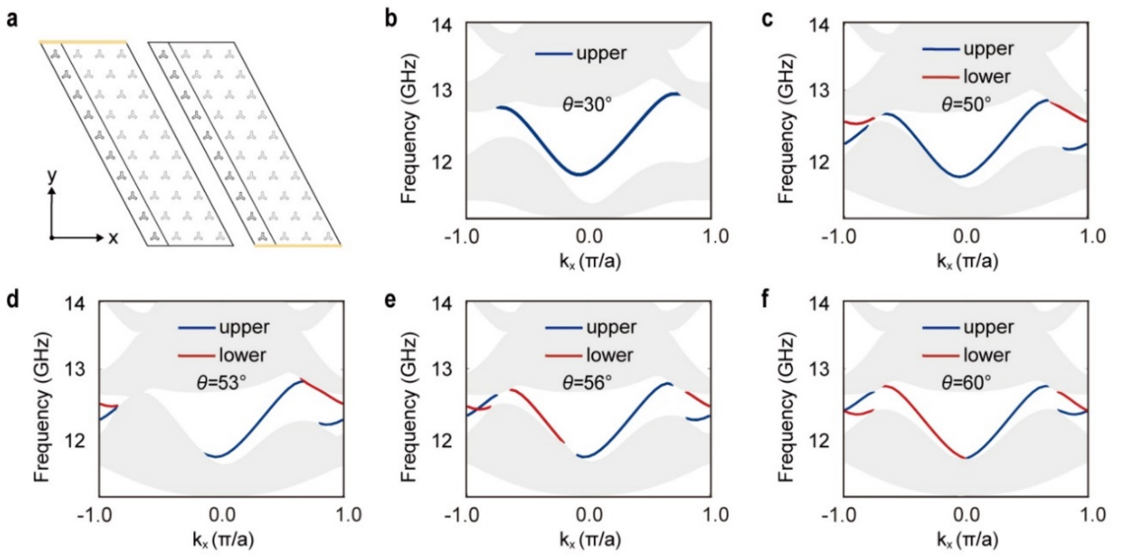

Supplementary Figure 4 | The evolution of the edge states. a Schematic of a supercell consisting of Y-shaped gyromagnetic photonic crystals cladded by the perfect electric conductor. **b-f** The eigenvalue spectrum of the supercell with **b** $\theta = 30^{\circ}$, **c** $\theta = 50^{\circ}$, **d** $\theta = 53^{\circ}$, **e** $\theta = 56^{\circ}$, **f** $\theta = 60^{\circ}$, respectively. Note that the edge state localized at upper and lower boundaries are colored in blue and red, respectively.

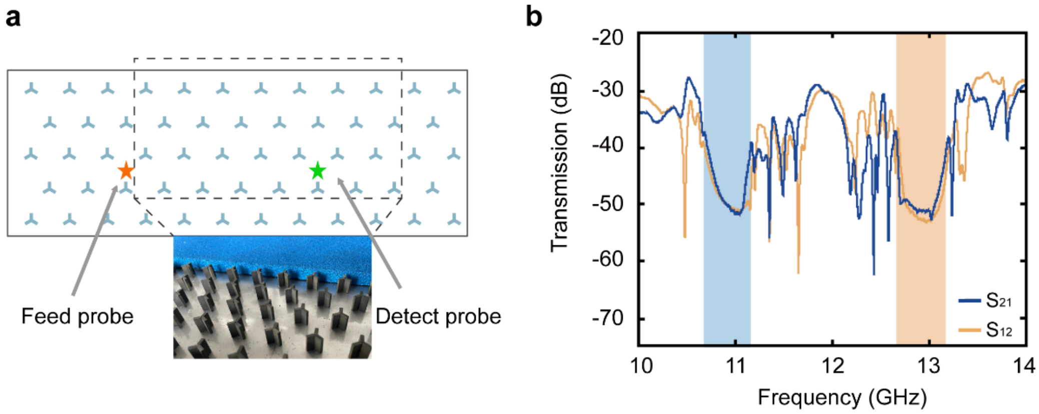

Supplementary Figure 5 | Experimental identification of the bulk band gap. a Schematic diagram of the experimental sample (5×12 unit cells) for valley edge states. The gray lines imply scattering boundaries. The feed probe is placed at the orange point on the left while the detected probe is placed at the right one inside the sample. The inset is the experimental sample. **b** The experimentally-measured bulk transmissions (S-parameter magnitudes). The frequency window of the QAH and VH phases are filled with blue and orange areas, respectively.

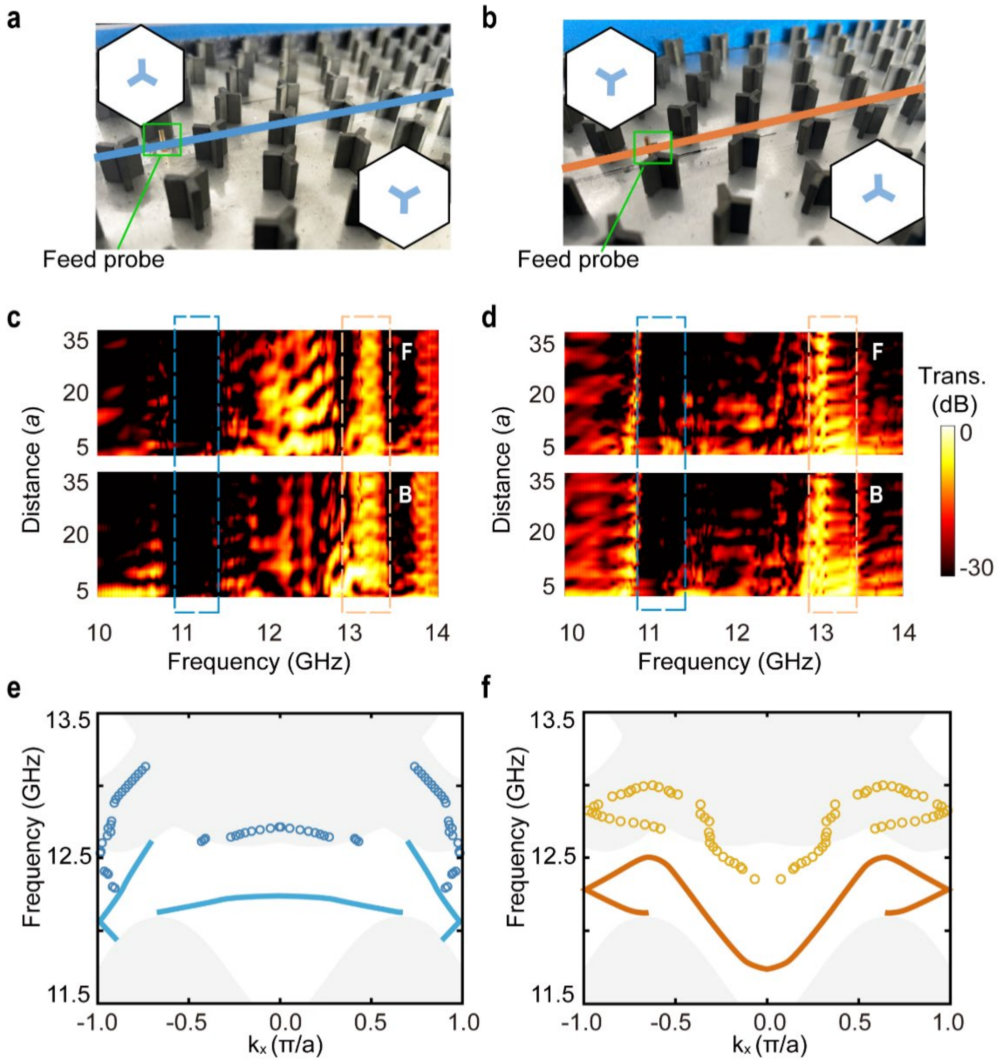

Supplementary Figure 6 | Observation of VH edge states with large valley Chern number. **a-b** Finite-sized samples (8×12 unit cells) of DW1(**a**) and DW2(**b**). Blue and orange lines represent different domain walls. The feed probe is framed in green. The air layer thickness is $d = 0.8$mm. **c-d** Scanned electric field distributions: frequency domains framed by dashed boxes display the lower gap (blue) and the frequency range of valley edge states (orange). The forward and backward transmissions are represented by '**F**' and '**B**', respectively. **e-f** The simulated (solid lines) and unprocessed experimental measured (hollow circles) valley edge dispersions for (c) DW1 and (d) DW2.

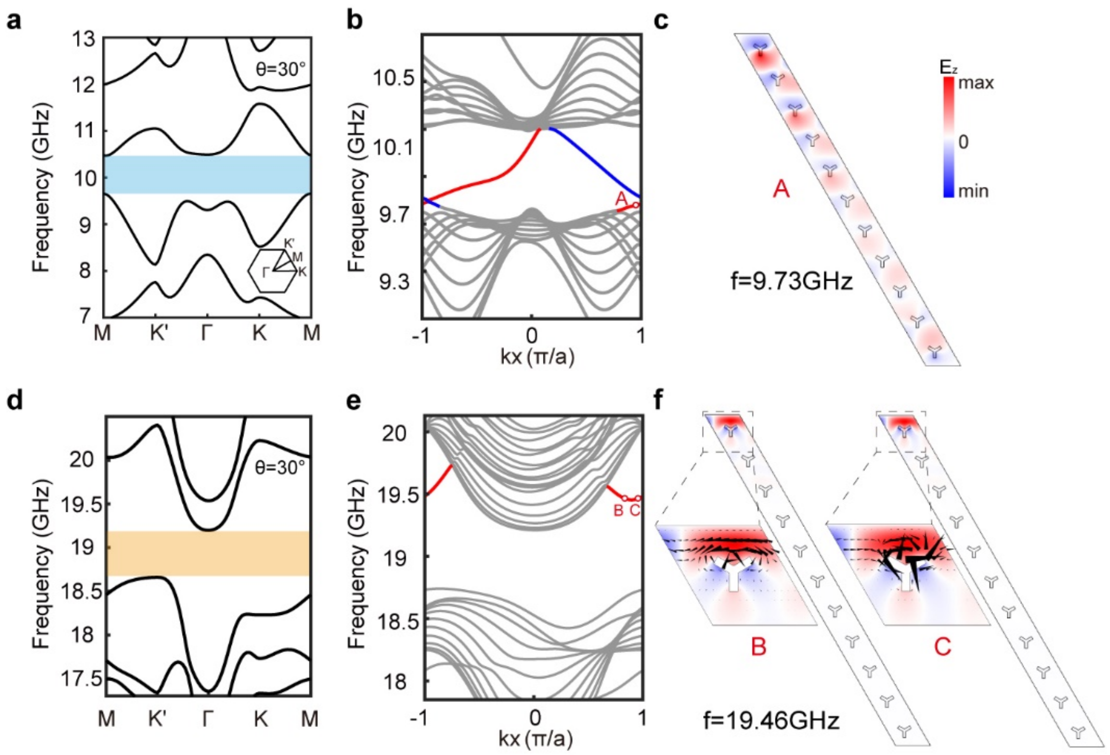

Supplementary Figure 7 | Hybrid topological photonic crystal with distinct topology in different band gaps. **a, d,** Bulk bands with a Chern gap and a valley Hall gap, respectively. **b, e,** The simulated edge dispersions corresponding to the topological band gaps in **a** and **d**, respectively. **c**, **f** The electric field patterns of the chiral edge states at frequency of 9.73GHz (marked by A in **b**), and the unbalanced valley edge states at frequency of 19.46GHz (marked by B and C in **e**).

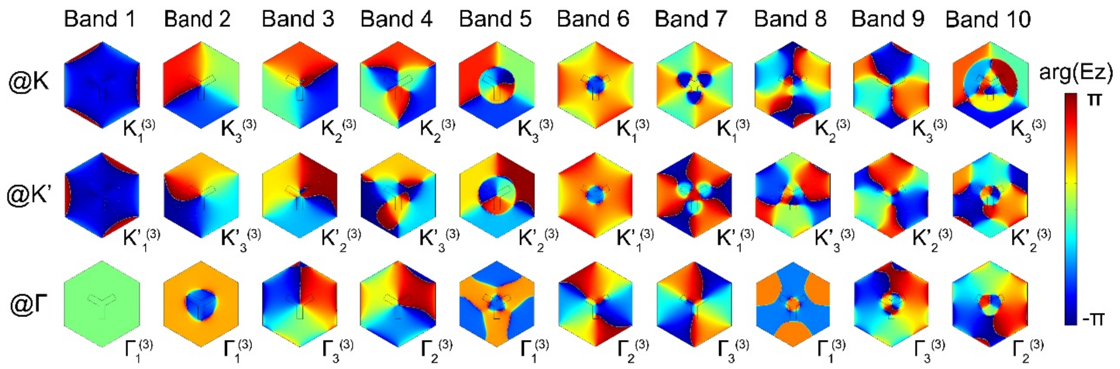

Supplementary Figure 8 | The phase distribution of the eigenstates. of the lowest ten bands that below gap IV at K, $K^{'}$, and Γ points, which gives the topological indicators.

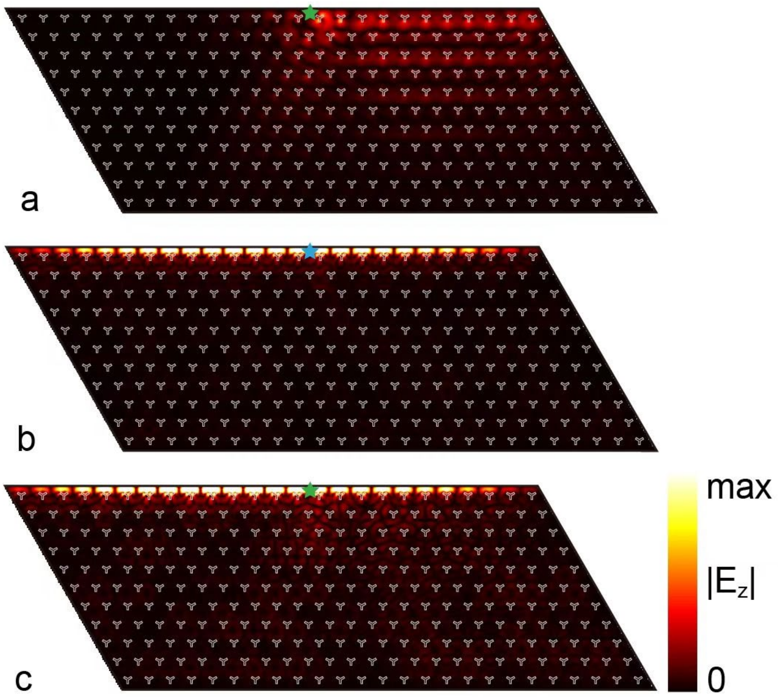

Supplementary Figure 9 | Nonlinear frequency conversion between the chiral edge states and valley Hall edge states. **a,** Simulated electromagnetic wave pattern excited by a point source with 9.73GHz (labeled by the green star) in the linear photonics domain (i.e., exciting the chiral edge states). **b,** Simulated electromagnetic wave pattern excited by a point source with 19.46GHz (labeled by the blue star) in the linear photonics domain (i.e., exciting the valley edge states). **c,** Simulated electromagnetic wave pattern excited by a point source with 9.73GHz (labeled by the green star) when the nonlinear effect is turned on.

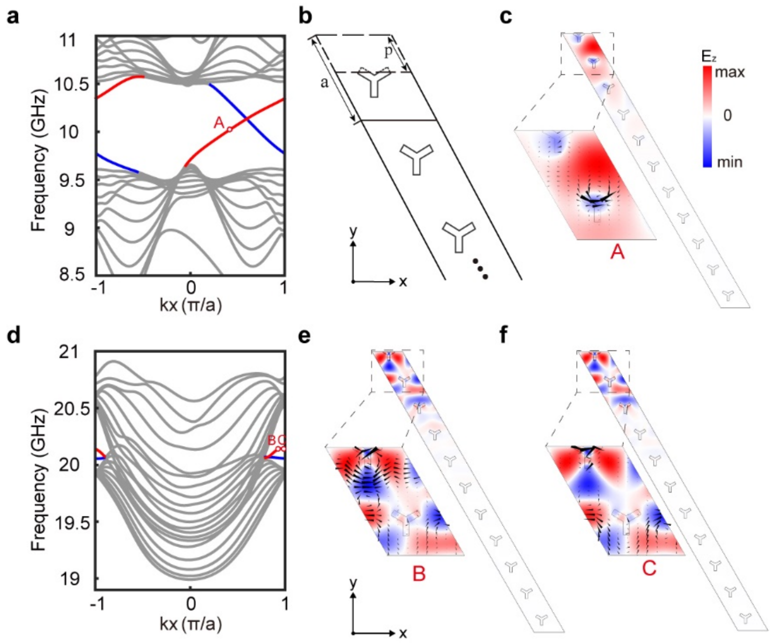

Supplementary Figure 10 | Hybrid topological photonic crystal with edge states satisfying frequency and phase matching for second-harmonic generation. a and **d,** The projected band structures at different frequency ranges. The red and blue curves correspond to the edge states for the upper and lower edge boundary of the supercell model in b, respectively. Here, A denotes an edge state in the lower band gap with a frequency $f_0 = 10.07$GHz, while B and C denote two edge states in the higher band gap with a frequency $f_2 = 2f_0 = 20.14$GHz. **b**, the schematic of the model with the upper edge termination is truncated at $p = 0.4a$. **c,e,f,** are the electric field distributions of the edge states A, B, and C, separately.

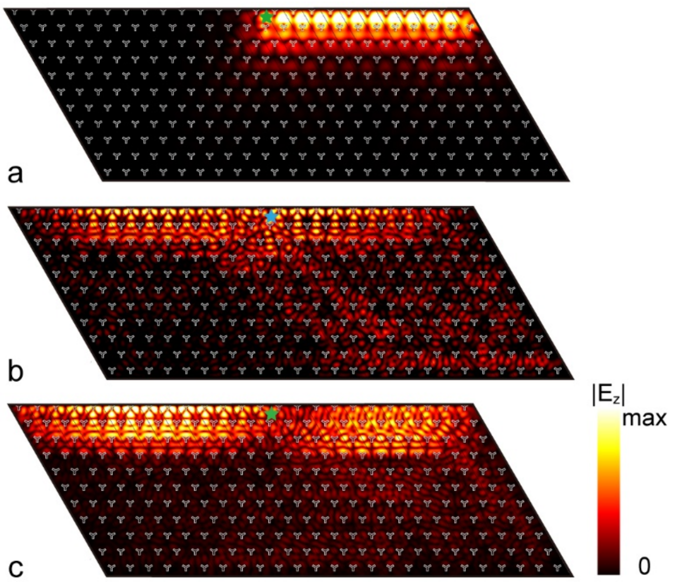

Supplementary Figure 11 | Efficient second harmonic frequency conversion in the edge channel of the hybrid topological photonic crystal. **a, b,** (Without nonlinear optical effects) Simulated electrical field distributions for the cases with a point source excitation at the frequency $f_0 = 10.07$GHz (source labeled by the green star) and $f_2 = 20.14$GHz (source labeled by the blue star), respectively. **c,** (With second-harmonic nonlinear optical effects) Simulated electrical field distribution for the case with a point source excitation at the frequency $f_0 = 10.07$GHz.

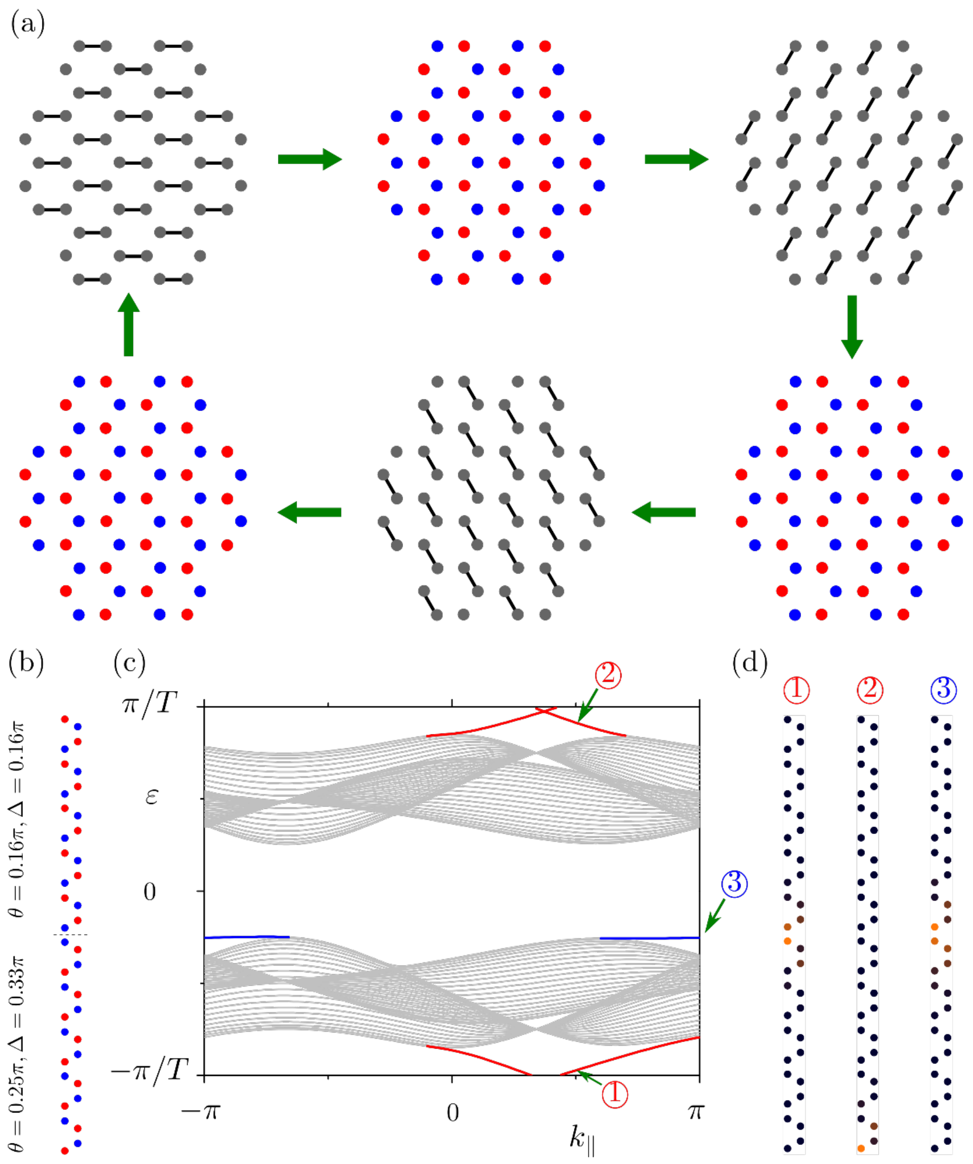

Supplementary Figure 12 | Hybrid topology in coupled waveguide arrays system via Floquet engineering. **a,** the time varying model which is composed of six steps. In odd steps the couplings strength is θ and in even steps the on-site potential difference is Δ. **b,** The strip structure with an interface between topological trivial state and HTPC state with different valley topology. The topological trivial state with $\theta = 0.16\pi$ and $\Delta = 0.16\pi$ has vanishing Chern number for both bands and the Chern number of HTPC state with $\theta = 0.25\pi$ and $\Delta = 0.33\pi$ is ± 1 for two bands. **c,** The quasi energy spectrum of the strip structure. The chiral edge states are colored by blue and the valley edge states are colored by red. **d,** The field distribution of chiral edge states and valley edge states marked in (**c**).

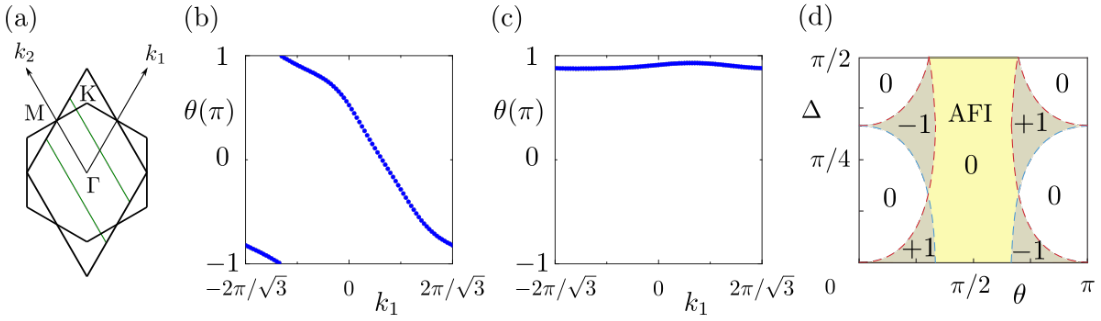

Supplementary Figure 13 | Numerical calculation of Chern numbers. a, Brillouin zone of the honeycomb lattice. **b,c,** The Berry phase as a function of k_1. The parameters are $\theta = 0.25\pi, \Delta = 0.33\pi$ for **b** and $\theta = 0.16\pi, \Delta = -0.16\pi$ for **c**. The Chern numbers are -1 and 0 for **b** and **c** respectively. **d,** Phase diagram. Chern numbers are marked. AFI is the anomalous Floquet topological insulator.

Supplementary Note 1: The calculation of band/gap Chern number of HTPC

To demonstrate the nontrivial band induced by the breaking of time-reversal symmetry (i.e., with an external magnetic field), we calculate the band Chern number via the integration of the Berry curvature, which is defined as

$$C_n = \frac{1}{2\pi}\iint_{BZ} \Omega_n(\mathbf{k})\, d^2\mathbf{k}. \tag{1}$$

Note that the integral is over the whole Brillouin zone (BZ) and $\Omega_n(\mathbf{k})$ is the Berry curvature of the *n*th band. By further summing the Berry curvature of bands below the gap of interest, we have the gap Berry curvature as

$$\Omega_g(\mathbf{k}) = \sum_n \Omega_n(\mathbf{k}). \tag{2}$$

Obviously, the integration of $\Omega_n(\mathbf{k})$ within the whole BZ gives rise to the gap Chern number C_g.

In order to verify the phase transition process of gap III, we adopt the efficient numerical algorithm reported in Ref. [1] to calculate the band (gap) Chern numbers. Supplementary Fig.2 presents the Berry curvature distributions of the lowest four bands associated with their Chern numbers for HTPC with $\theta = 0^\circ, 30^\circ, 90^\circ$, which represent three different typical cases of HTPC. For all these three cases, it is seen that the Berry curvature distributions of the first, second, and third bands are almost the same. The integration of band Berry curvature of the first, second, and third band give $C_1 = 0, C_2 = -1, C_3 = 2$, respectively. In contrast, the Berry curvature distribution of the fourth band make an evident difference in these three cases, which the Chern number gives $C_4 = 1$ for HTPC with $\theta = 0^\circ$, $C_4 = -1$ for HTPC1 ($\theta = 30^\circ$) , and $C_4 = -1$ for HTPC2 ($\theta = 90^\circ$). By further summing the Berry curvature of bands below the gap of interest, we also present the Berry curvature distributions of gaps II and III in Supplementary Fig. 3. For the gap II of HTPC with $\theta = 0^\circ, 30^\circ, 90^\circ$, it is evident that Berry curvature has positive values, indicating that the gap II is of nontrivial topological nature (see Supplementary Fig. 3a-3c). A similar phenomenon can be found in gap III of HTPC with $\theta = 0^\circ$. However, for the gap III of HTPC with $\theta = 30^\circ$ and $\theta = 90^\circ$, the Berry curvatures have both positive and negative values, in contrast to the former

situation. Further integration of the Berry curvature of gap III gives $C_{III} = 0$ for both HTPC1 ($\theta = 30^{\circ}$) and HTPC2 ($\theta = 90^{\circ}$).

Supplementary Note 2：The derivation of valley Chern number

In the maintext, we claim that the valley Hall gap has a large valley Chern number, which is induced by gapping the unpaired quadratic Dirac point. Since the numerical calculation of the valley Chern number is not accurate, here we derive the valley Chern number via an analytical model. As an illustration, we consider the effective Hamiltonian of HTPC1 around the K valley, which is induced by gapping quadratic Dirac point, and hence could be written in the form of massive Dirac Hamiltonian,

$$\begin{aligned} H(\mathbf{k}) &= \mathbf{h}(\mathbf{k}) \cdot \boldsymbol{\sigma} \\ &= v_D\left[\left(k_x^2 - k_y^2\right)\sigma_x - 2k_x k_y \sigma_y\right] + M\sigma_z, \end{aligned} \tag{3}$$

where v_D is the Dirac velocity, $\mathbf{k} = (k_x, k_y)$ measures the momentum deviation from the K valley, M refers to the mass term induced by the C_{3v} and/or $\mathcal{T}$ symmetries, and $\sigma_i (i = x, y, z)$ are Pauli matrices operating on the orbital degree of freedom. The Berry curvature is then given by[2]

$$\begin{aligned} \Omega(\mathbf{k}) &= \frac{\mathbf{h}}{2h^3} \cdot \left(\partial_{k_x}\mathbf{h} \times \partial_{k_y}\mathbf{h}\right) \\ &= \frac{-2Mv_D^2\mathbf{k}^2}{(v_D^2\mathbf{k}^4 + M^2)^{3/2}}. \end{aligned} \tag{4}$$

Henceforth, the valley Chern number can be attained by integrating the Berry curvature over the whole 2D space, which gives by

$$\begin{aligned} C_K &= \frac{1}{2\pi}\int \frac{-2Mv_D^2\mathbf{k}^2}{(v_D^2\mathbf{k}^4 + M^2)^{\frac{3}{2}}} d^2\mathbf{k} \\ &= \frac{1}{2\pi}\int_0^{\infty} \frac{-2Mv_D^2k^2}{(v_D^2k^4 + M^2)^{\frac{3}{2}}} (2\pi k)dk \\ &= -\frac{1}{2}\int_0^{\infty} M(v_D^2k^4 + M^2)^{-\frac{3}{2}} d(v_D^2k^4). \end{aligned} \tag{5}$$

Let $v_D^2k^4 = x$, we have

$$C_K = -\frac{1}{2}\int_0^{\infty} M(x+M^2)^{-\frac{3}{2}}dx$$
$$= M(x+M^2)^{-\frac{1}{2}}\Big|_0^{\infty}$$
$$= -\mathrm{sgn}(M). \quad (6)$$

Remarkably, we find that the valley Chern number induced by gapping a quadratic Dirac point is an integer number, in strong contrast to the common perception that the valley Chern number is a half-integer.

As we have pointed out in the maintext, m_v and m_T are the mass terms induced by breaking the C_{3v} (through rotation) and $\mathcal{T}$ (through external magnetic field) symmetries, separately. Note that in our work the external magnetic field gives $m_T < 0$, and the rotation operation gives $m_v < 0$ when $\theta \in (0^\circ, 60^\circ)$, and $m_v > 0$ when $\theta \in (60^\circ, 120^\circ)$. Hence, the total mass term induced by breaking both C_{3v} and $\mathcal{T}$ around the K valley is $C_K = -sgn(m_T - m_v)$ while that around K' valley is $C_{K'} = -sgn(m_T + m_v)$.

Supplementary Note 3: The evolution of the edge states under the perfect electric conductor boundary condition

To manifest the phase transition between QAH and VH, we further examine the evolution of the edge states under the perfect electric conductor boundary condition. As shown in Supplementary Fig. 4a, we first construct a supercell consisting of 10 HTPC terminated by perfect electric conductors. Note that the supercell is expanded into 4 periods in the x-direction for clarity. We then perform the eigenvalue calculation of the supercell with $\theta = 30^\circ, 50^\circ, 53^\circ, 56^\circ, 60^\circ$, respectively, which represent several typical topological phases, i.e., the gap III is of VH phase when $\theta = 30^\circ, 50^\circ$, while of QAH phase when $\theta = 53^\circ, 56^\circ, 60^\circ$. As is expected, the projected band structures associated with gapped edge states in Supplementary Figs. 4b and 4c indicate that gap III is of VH phase, while the projected band structures associated with gapless edge states in Supplementary Figs. 4d-4f indicate that gap III is of QAH phase. Note that the edge state localized at upper and lower boundaries are colored in blue and red, respectively. Hence, the edge states evolve from the gapped to gapless dispersions,

which provide direct evidences of phase transition from VH to QAH phase during the rotation tuning process.

Moreover, we notice under the perfect electric conductor boundary condition, the valley edge dispersion can be effectively tuned by rotation angle. As shown in Supplementary Figs. 4b and 4c, the HTPC with $\theta = 30^{\circ}$ only exhibits the upper edge state, while the HTPC with $\theta = 50^{\circ}$ hosts both upper and lower edge states. During the increase of rotation angle, the lower edge states gradually emerged into the bulk states and eventually become the edge states when $\theta = 30^{\circ}$. Although the valley Chern number of HTPC1 is nonzero, the HTPC with the rigid boundary does not strictly contribute a bulk-edge correspondence, leading to the boundary-configured valley edge states [3,4].

Supplementary Note 4: Identification of the bulk gap

Before implement the measurement of the edge states, we first identified the full band gap of the HTPC2. As shown in Supplementary Fig. 5a, the HTPC2 composed of 5×12 cells are placed in a paralleled plate waveguide (PPW) and surrounded with EM absorber sponges. Both feed and detect probes (marked as orange and green stars) are laid in the HTPC with a distance of $5a$. The experimentally-measured bulk transmissions are displayed in Supplementary Fig. 5b. The transmission coefficients S21 and S12 have the similar curves, thus showing reciprocity. Under the external magnetic field of 700 Oe, two band gaps appear in the frequency range of 10.61-11.25 GHz and 12.68-13.18 GHz, corresponding to topological QAH and VH phases, respectively, which is being consistent with the frequency ranges framed by dotted boxes in Figs. 3g and 3h.

Supplementary Note 5: Observation of valley Hall edge states

In view of one-dimensional near field scanning, an inevitable air layer between the plate of PPW and the sample is necessary for detect probe sliding, which makes the effective refraction lower and causes bands shift. Usually, the larger sample needs thicker air layer and the thicker air layer brings larger frequency shift[5]. When the air

layer thickness $d = 0.8$mm which is utilized in our VH edge state experiments, there exist frequency shifts in the measured valley edge states compared with the simulation ones. The two samples with DW1 and DW2 shown in Supplementary Figs. 5a and b are placed in the PPW with a fixed feed probe and a sliding detect probe. We set stepped displacement as 4mm (about $a/5$). Supplementary Figures 5c and 5d describe the mapped electric field distributions along the domain wall under 700 Oe. For both DW1 and DW2, the EM wave is forbidden in QAH gap (marked in blue dashed box), showing no edge mode excited in the QAH gap. As a comparison, in the VH gap (orange dashed box), EM wave can propagate both forward and backward along the edge but with different profiles, showing the property of the VH edge state. The valley edge dispersions in simulations and experiments for two samples are described in Supplementary Fig. 5e and 5f. The frequency difference between the experimental and simulated valley edge states is about 500M, due to the inevitable air layer ($d = 0.8$mm) in the experiments.

Supplementary Note 6: Hybrid topological photonic crystals with nonlinear effect

In this section, we discuss the physical phenomenon when adding the nonlinear effect into the hybrid topological systems. To demonstrate the nonlinear optical process, e.g., second-harmonic generation, it is necessary to find two edge states with distinct topologies, which satisfies a double frequency relationship. Hence, we plot the band structures of HTPC1 in the range of 7-13 GHz and 17-21 GHz that consisting complete band gaps (colored by blue and orange) in Supplementary Fig. 7a and 7d, respectively. For simplicity, we term the band gap within 7-13 GHz (17-21 GHz) as gap L (U). Since we have demonstrate that the gap L is a Chern gap in the maintext, here we utilize the symmetry indicators to characterize the topological property of the gap U.

Following Ref. [6], the topological crystalline index can be expressed by the full set of the C_3 eigenvalues at the high-symmetry points (HSPs). For an HSP denoted by the symbol Π, the C_3 eigenvalues can only be $\Pi_n = e^{i2\pi(n-1)/3}$ with $n = 1,2,3$. Here, the HSPs include Γ, K, and K' points. Hence, for the HTPC with C_3 symmetry, the topological indices that describe the band topology is given by

$$\chi = \{[K]_n, [K']_n\} = \{\#K_n - \#\Gamma_n, \#K'_n - \#\Gamma_n\} \;\; n = 1,2, \tag{7}$$

where $\#K_n$, $\#K'_n$, and $\#\Gamma_n$ are the numbers of bands below the band gap with the C_3 symmetry eigenvalue K_n, K'_n, and Γ_n at the K, K', and Γ points, respectively. In this scheme, the point is taken as the reference point to get rid of the redundance. Any nonzero indicates a topological band gap that is adiabatically disconnected from the trivial atomic insulator. In Supplementary Fig. 8, we present phase distribution of the eigenstates of the lowest ten bands that below gap U at K, K', and Γ points, where the C_3 eigenvalues are also labeled. Therefore, the topological indices of the gap U is given by

$$\chi = \{[K]_1, [K']_1, [K]_2, [K']_2\} = \{-1, -1, 0, 1\}, \tag{8}$$

which indicate the gap U is of nontrivial topological property. Note that in our case, the characterization of the valley topology via the symmetry indicator is equivalent to the Berry curvature calculation, hence, we remark that the gap U is of valley topology.

We then present in Supplementary Figs. 7b and 7e the corresponding photonic band structures of a supercell that consisting of 11 HTPC1 unit-cells terminated by perfect electric conductors. As shown in Supplementary Fig. 7b, two edge branches go across the whole gap II whose typical electric field patterns at frequency of 9.73GHz is shown in Supplementary Fig. 7c. Meanwhile, unbalanced valley Hall edge states (Supplementary Fig. 7e) emerge in gap IV whose electric field pattern at frequency of 19.46GHz is shown in Supplementary Fig. 7f with different Poynting vector directions. We remark that the gapless edge state in gap II originates from its QAH topology nature while the gapped edge state II is of its valley topology nature.

We now investigate the nonlinear frequency conversion process via the edge modes that indicated in Supplementary Figs. 7c and 7f. The full-wave dynamics of the nonlinear interaction of chiral edge states and valley edge states were determined numerically using the commercial software COMSOL MULTIPHYSICS. To simulate the nonlinear frequency mixing processes in COMSOL, we defined two "Electromagnetic Waves, Frequency Domain" models: one for the fundamental frequency ω_0 and one for the second harmonic frequency Ω_2. These two models are

coupled using a "Polarization" feature added to each of the models. We assumed that for second harmonic generation, the nonlinear susceptibilities are diagonal tensors with the diagonal elements being χ_2. Hence, for second harmonic generation, the nonlinear polarizations at the fundamental frequency and second harmonic are

$$P_{NL,z}^{\omega_0} = 2\chi_2 E_{2z} E_{1z}^*, \tag{9}$$

$$P_{NL,z}^{\Omega_2} = \chi_2 E_{1z}^2. \tag{10}$$

As an illustration, we consider HTPCs made of homogeneous and isotropic nonlinear material with a scalar nonlinear second-order susceptibility of $\chi_2 = 10^{-21}\mathrm{C\,V^{-2}}$. The pump electric field E_{1z} is induced by an external point source, where E_{2z} by the nonlinear polarization at the second harmonic, generated by E_{1z}. The main results are summarized in Supplementary Fig. 9.

As shown in Supplementary Fig. 9a, a typical chiral edge mode is excited by a point source at the frequency of $f_1 = 9.73$GHz. As a comparison, at the frequency of $f_2 = 19.46$GHz both edge and bulk modes can be excited (see Supplementary Fig. 9b). Under the point excitation in the edge, the electrical field distributions mainly at the boundary with strong localization and obvious asymmetry and little into the sample at $f_2 = 2f_1 = 19.4$GHz, which matches well with the band structure. After introducing nonlinear polarizations, the electric field E_{2z} in Supplementary Fig. 9c distributes more into the bulk than that in Supplementary Fig. 9b, indicating that these two edge modes are indeed nonlinearly interacting via the second harmonic generation, which demonstrates nonlinear effect generates new physical phenomenon.

Since the phase matching condition plays a key role on the realization of the nonlinear effect, here we also achieve the quasi-phase-matching condition to enhance the nonlinear optical effect on the manipulation of the edge propagation of photons by tailoring the dispersion of the edge states. For this purpose, we tailor the edge dispersions by cutting part of the unit-cells at the edge boundary. This is illustrated in Supplementary Fig. 10b with a geometry parameter $p = 0.4a$ where a =21mm is the lattice constant. The resultant dispersion of the topological edge states in the quantum anomalous Hall photonic band gap around 10GHz is shown in Supplementary Fig. 10a,

while the electric field profile of a specific edge state (labeled the red-letter A) is shown in Supplementary Fig. 10c. The dispersion of the edge states in the valley Hall photonic band gap is shown in Supplementary Fig. 10d.

We consider here the second-harmonic generation between the edge state A in Supplementary Figs. 10a and 10c with the fundamental frequency $f_0 = 10.07$GHz and the wavevector $k_0 = 0.495\frac{\pi}{a}$ and the resonant second harmonic edge states B and C in Supplementary Figs. 10d, 10e, and 10f with a frequency $f_2 = 20.14$GHz. The edge states B and C have the wavevectors $k_{21} = 0.935\frac{\pi}{a}$ and $k_{22} = \frac{\pi}{a}$, respectively. Interestingly, the edge states A and B have positive group velocity, while the edge state C has negative group velocity. The wavevector mismatch for the edge states B and C are small, $\Delta k_1 = k_{21} - 2k_0 = -0.055\frac{\pi}{a}$ and $\Delta k_2 = k_{22} - 2k_0 = 0.01\frac{\pi}{a}$, both showing quasi-phase-matching.

The edge state A can be visualized by a point source excitation at the edge boundary with the frequency f_0 (see Supplementary Fig. 11a), showing elegantly the unidirectional photonic edge propagation (right-going). On the other hand, the edge states B and C can both excited by a point source at the same position with frequency f_2 (Supplementary Fig. 11b), showing bidirectional photonic edge propagation with asymmetric features (i.e., the right-going signal decays faster than the left-going signal). With second-harmonic nonlinear effects and exciting at the frequency f_0 at the same position, the photonic energy flow along the edge channel now switches from unidirectional to bidirectional (Supplementary Fig. 11c). The simulation results in Supplementary Fig. 11c show comparable left-going and right-going photonic energy flow which is an evidence that the second-harmonic frequency conversion is efficient. We remark that the above phenomenon has not yet been found before, since in the previous studies, the topological edge states in different band gaps propagate in the same direction[7].

With the above calculations and discussions, we demonstrate through a concrete example that the phase-matching condition can be satisfied to achieve efficient second-harmonic frequency conversion among the edge states in distinct topological band gaps.

Such frequency conversion can be used to manipulate photonic energy flows in the topological edge channel which is a new degree of freedom that can be promising for future topological photonics.

Supplementary Note 7: Photonic Floquet hybrid topological insulators

In the maintext, the hybrid topological system is demonstrated by using gyromagnetic materials, which have weak magneto-optical responsive in the optical frequencies. However, we remark that the results still can be extended to optical frequencies since the design principle of the hybrid topological system merely requires for the breaking of both $\mathcal{P}$ and $\mathcal{T}$ symmetries. To this end, we implement the Floquet engineering to realize the hybrid topology in coupled waveguide arrays, which offers a possible scheme to extend to optical frequencies.

The time varying model is shown in Supplementary Fig. 12a, which contains six steps. The time-dependent Hamiltonian of the system can be described by,

$$H_{\boldsymbol{k}}(t) = \sum_{m=1,3,5} \theta(t)\left(e^{i\boldsymbol{b}_m\cdot\boldsymbol{k}}\sigma^+ + \mathrm{H.c.}\right) + \Delta(t)\sigma_z, \tag{11}$$

where $\theta(t)$ is set to be θ at odd steps, and zero at even steps; $\Delta(t)$ equals Δ at even steps, and zero at odd steps; $\sigma^{\pm} = \left(\sigma_x \pm i\sigma_y\right)/2$, where $\sigma_{x,y,z}$ are Pauli matrices; the vectors b_{2n+1} are given by $b_1 = \left(\frac{a}{\sqrt{3}}, 0\right), b_2 = \left(-\frac{a}{2\sqrt{3}}, -\frac{a}{2}\right), b_3 = \left(-\frac{a}{2\sqrt{3}}, \frac{a}{2}\right)$, where a is the lattice constant. The spectrum of the system can be obtained by solving eigen equation of Floquet operator,

$$U_F|\phi\rangle = e^{-i\varepsilon T}|\phi\rangle, \tag{12}$$

here the Floquet operator is defined as $U_F \equiv \mathcal{T}\exp\left[-i\int_{t_0}^{t_0+T} H(\tau)\,d\tau\right]$, where $\mathcal{T}$ is the time-ordering operator. Different from the static system, here we study the quasi-energy ε, which has a periodicity of $2\pi/T$.

From the model, we notice the time reversal symmetry of the system is broken due to the periodically driving and the parity symmetry is also broken when Δ is nonzero. Besides, the lattice sites are arranged in a honeycomb lattice so that the model is suitable for studying valley topology and quantum anomalous Hall physics, and may support

hybrid topological states. The phase diagram of the system is shown in Supplementary Fig. 13d, which contains topological trivial state with vanishing Chern number, Chern insulator with Chern number ± 1 and anomalous Floquet topological insulator also with vanishing Chern number but supports chiral edge state. We study the strip structure shown in Supplementary Fig. 12b which contains the interface between topological trivial state (upper) and hybrid topological state (lower) with different valley topology. The Chern number is -1 for the first bulk band of HTPC which can be obtained from Berry phase calculation in Supplementary Fig. 13b, we can see the winding of Berry phase as function of k_1 is -1. The Chern number is 0 for topological trivial state whose Berry phase winding as function of k_1 is 0 as shown in Supplementary Fig. 13c. The quasi-energy spectrum of the strip structure is shown in Supplementary Fig. 12c. There are two bulk bands due to the bipartite nature of the system and two band gaps, one at quasi energy 0 and the other at quasi energy π/T. The π/T gap belongs to quantum anomalous Hall phases and supports chiral edge state and chiral interface states colored by red. The 0 gap of topological trivial state and HTPC state belongs to different quantum valley Hall phases (the sign of Δ is opposite) so that there are valley interface states colored by blue. The field distribution of chiral edge states, chiral interface state and valley interface states are shown in Supplementary Fig. 12d. We notice the field of the chiral edge state is localized at the lower edge which can be treated as the interface between HTPC and vacuum, the fields of the chiral interface state and valley interface state are localized at interface. It is worthy to mention that, although here we propose a time varying system, in the real optical experiment we can use the coupled helical waveguides arrays which treat one spatial direction as time. Such system has been used to study photonic Floquet topological insulator[8,9], photonic valley topological insulator[10], photonic anomalous Floquet topological insulator[11–14] and photonic anomalous Flouqet higher-order topological insulator[15] in optical region. Our proposal here provides a new member to the family of topological insulator in optical region.

References:

1. Fukui, T., Hatsugai, Y. & Suzuki, H. Chern Numbers in Discretized Brillouin Zone: Efficient Method of Computing (Spin) Hall Conductances. *J. Phys. Soc. Jpn.* **74**, 1674–1677 (2005).
2. Xiao, D., Chang, M.-C. & Niu, Q. Berry phase effects on electronic properties. *Rev. Mod. Phys.* **82**, 1959–2007 (2010).
3. Wei, G. *et al.* Boundary configured chiral edge states in valley topological photonic crystal. *Opt. Lett.* **47**, 3007 (2022).
4. Fan, X., Xia, T., Qiu, H., Zhang, Q. & Qiu, C. Tracking Valley Topology with Synthetic Weyl Paths. *Phys. Rev. Lett.* **128**, 216403 (2022).
5. Xie, B.-Y. *et al.* Visualization of Higher-Order Topological Insulating Phases in Two-Dimensional Dielectric Photonic Crystals. *Phys. Rev. Lett.* **122**, 233903 (2019).
6. Benalcazar, W. A., Li, T. & Hughes, T. L. Quantization of fractional corner charge in C_n-symmetric higher-order topological crystalline insulators. *Phys. Rev. B* **99**, 245151 (2019).
7. Lan, Z., You, J. W. & Panoiu, N. C. Nonlinear one-way edge-mode interactions for frequency mixing in topological photonic crystals. *Phys. Rev. B* **101**, 155422 (2020).
8. Rechtsman, M. C. *et al.* Photonic Floquet topological insulators. *Nature* **496**, 196–200 (2013).
9. Maczewsky, L. J. *et al.* Fermionic time-reversal symmetry in a photonic topological insulator. *Nat. Mater.* **19**, 855–860 (2020).
10. Noh, J., Huang, S., Chen, K. P. & Rechtsman, M. C. Observation of Photonic Topological Valley Hall Edge States. *Phys. Rev. Lett.* **120**, 063902 (2018).
11. Leykam, D., Rechtsman, M. C. & Chong, Y. D. Anomalous Topological Phases and Unpaired Dirac Cones in Photonic Floquet Topological Insulators. *Phys. Rev. Lett.* **117**, 013902 (2016).
12. Maczewsky, L. J., Zeuner, J. M., Nolte, S. & Szameit, A. Observation of photonic anomalous Floquet topological insulators. *Nat Commun.* **8**, 13756 (2017).
13. Mukherjee, S. *et al.* Experimental observation of anomalous topological edge modes in a slowly driven photonic lattice. *Nat Commun.* **8**, 13918 (2017).

14. Pyrialakos, G. G. *et al.* Bimorphic Floquet topological insulators. *Nat. Mater.* **21**, 634–639 (2022).
15. Zhu, W., Chong, Y. D. & Gong, J. Floquet higher-order topological insulator in a periodically driven bipartite lattice. *Phys. Rev. B* **103**, L041402 (2021).
